# Serum S-100β and NSE levels after off-pump versus on-pump coronary artery bypass graft surgery

**DOI:** 10.1186/s12872-015-0050-0

**Published:** 2015-07-16

**Authors:** Lei Zheng, Qing-Ming Fan, Zhen-Yu Wei

**Affiliations:** Department of Cardiovascular Surgery, Yantai Yuhuangding Hospital, No.20 Yuhuangding East Road, Yantai, 264000 P.R. China

**Keywords:** S-100β, NSE, Coronary heart disease, Coronary artery bypass grafting, Meta-analysis

## Abstract

**Background:**

We aimed to evaluate serum levels of S-100 beta (S-100β) and neuron specific enolase (NSE) in patients with coronary heart disease (CHD) after off-pump versus on-pump coronary artery bypass graft (CABG) surgery.

**Methods:**

The PubMed (~2013) and the Chinese Biomedical Database (CBM) (1982 ~ 2013) were searched without language restrictions. After extraction of relevant data from selected studies, meta-analyses were conducted using STATA software (Version 12.0, Stata Corporation, College Station, Texas USA). Possible sources of heterogeneity were examined through univariate and multivariate meta-regression analyses and verified by Monte Carlo Simulation.

**Results:**

Eleven studies with a total of 411 CHD patients met the inclusion criteria. Our meta-analysis showed no significant difference in serum S-100β and NSE levels between the on-pump group and the off-pump group before surgery. In the on-pump group, there was a significant difference in serum S-100β levels of CHD patients between before and after surgery, especially within the first 24 h after surgery. Furthermore, in the on-pump group, there was a significant difference in serum NSE levels of CHD patients between before and after surgery, particularly at 0 h after surgery. In the off-pump group, there was an obvious difference in serum S-100β levels between before and after surgery, especially within 24 h after surgery. Our results also demonstrated that serum S-100β and NSE levels of CHD patients in the on-pump group were significantly higher than those of patients in the off-pump group, especially within 24 h after surgery.

**Conclusions:**

Our findings provide empirical evidence that off-pump and on-pump CABG surgeries may increase serum S-100β and NSE levels in CHD patients, which was most prominent within 24 h after on-pump CABG surgery.

## Background

As a major public health issue worldwide, coronary heart disease (CHD) is the primary cause of disability and death in the developed countries and is among the leading causes of disease burden in low-and middle-income countries [1, 2]. Evidence has revealed that the prevalence of CHD in persons aged 20 years or older was estimated to be 6.4 % (15.4 million) in the US in 2010, and 386,324 cases of CHD-related deaths were reported in 2009 [3]. Nowadays, three therapeutic options are generally used for patients with CHD, including medical treatment with drugs, coronary interventions such as angioplasty and coronary stent implantation, and coronary artery bypass grafting (CABG) surgery [4, 5].

CABG surgery is a surgical procedure most commonly performed to relieve angina and reduce the risk of death from CHD [6]. The CABG surgery has significantly changed over the years, from traditional surgical operations using cardiopulmonary bypass (on-pump CABG) to a newer approach in cardiovascular surgery (off-pump CABG), both of which are primarily designed to improve the outcomes in CHD patients [7, 8]. Although the operative mortality in CABG surgeries has decreased dramatically, the rate of neurologic complications remains unacceptably high; for example, neurological injury is a major perioperative risk in these patients [9, 10]. Unfortunately, the postoperative brain damage is difficult to diagnose early and mainly based on the observation of specific brain injury markers [11]. Recently, it has been reported that the cerebral biomarkers such as S-100 beta (S-100β) and neuron specific enolase (NSE) may serve as biomarkers to reflect brain damages in cardiac surgery [12]. S-100β protein, a specific protein originating from the brain, has been found in the cytosol of both glial and Schwann cells, chondrocytes and adipocytes, having both intracellular and extracellular neurotropic and also neurotoxic functions [13, 14]. Low physiological concentrations of S-100β could protect neurons against apoptosis, stimulate neurite outgrowth and astrocyte proliferation, whereas S-100β at high concentrations may result in neuronal death and exhibit properties of a damage-associated molecular pattern protein [15, 16]. In addition, elevated levels of S-100β might accurately reflect the existence of neuropathological conditions, including neurodegenerative diseases and neuronal injury [17, 18]. NSE has also been suggested to act as a specific serum marker for neuronal damage, which is mainly found in neuronal cells, especially in mature neurons of the central nervous system, and is not secreted; and thus, increased NSE in cerebrospinal fluid or blood may reflect postoperative cognitive dysfunction or structural damage to neuronal cells [19, 20]. Therefore, serum S-100β and NSE levels measured before and after on-pump and off-pump CABG could potentially be diagnostic of ongoing cerebral damage associated with these surgical procedures [21]. To date, evidence supports that both on-pump and off-pump CABG are associated with increased serum levels of NSE and S-100β, but the off-pump CABG exhibits relatively lower serum S-100β protein and NSE levels, suggesting that the off-pump CABG has less influence or impairment on neurocognitive functions in comparison to the on-pump CABG [22, 23]. However, contradictory results have also been reported in the literature. Therefore, we performed this meta-analysis aiming to evaluate serum S-100β and NSE levels in CHD patients after off-pump versus on-pump CABG surgery.

## Methods

### Literature search and selection criteria

The PubMed (~2013) and the Chinese Biomedical Database (CBM) (from 1982 to 2013) were searched without language restrictions. The keywords and MeSH terms applied in combination with a highly sensitive search strategy were: (“S100 calcium binding protein beta subunit” or “nerve tissue protein S100b” or “neurotrophic protein S100beta” or “S-100β” or “S100beta protein” or “S100beta”) and (“phosphopyruvate hydratase” or “2-phospho-D-glycerate hydrolase” or “NSE” or “neuron-specific enolase” or “nervous system specific enolase” or “muscle specific enolase”) and (“coronary artery bypass” or “coronary artery bypass grafting” or “CABG” or “on-pump coronary artery bypass” or “off-pump coronary artery bypass” or “on- and off- coronary artery bypass”). Moreover, a manual search based on the references lists of the searched articles was also carried out to identify other potential articles.

The eligibility criteria for the inclusion of studies in this meta-analysis were as follows: (1) the study must report serum S-100β and NSE levels in CHD patients after off-pump versus on-pump CABG surgery; (2) all patients must have confirmed the diagnostic criteria for CHD; (3) the study must supply sufficient information on serum levels of S-100β and NSE. Studies that did not meet the inclusion criteria were excluded. In case those authors published the same subjects in several studies, the most recent study or the study with largest sample size was selected.

### Data extraction and methodological assessment

Using a standardized data extraction form, two authors independently extracted the following information from the studies included: publication year of article, geographical location, language of publication, surname of the first author, sample size, the source of the subjects, design of study, follow-up time, detection method, serum levels of S-100β and NSE, etc. Methodological quality assessment was carried out respectively by two authors through the Newcastle-Ottawa Scale (NOS) criteria [24]. Three aspects were included in the NOS criteria: (1) subject selection: 0 ~ 4; (2) comparability of subject: 0 ~ 2; (3) clinical outcome: 0 ~ 3. The range of NOS scores is from 0 to 9; and a score of ≥ 7 represents a high quality.

### Statistical analysis

The STATA statistical software (Version 12.0, Stata Corporation, College Station, TX, USA) was applied for our meta-analysis. Standardized mean difference (SMD) with the corresponding 95 % confidence intervals (95 % CI) was calculated. In addition, the *Z* test was conducted for estimation of the statistical significance of pooled SMDs. Heterogeneity among studies was estimated by the Cochran’s *Q*-statistic and *I*^*2*^ tests [25]. If the *Q*-test showed a *P* < 0.05 or the *I*^*2*^ test showed > 50 %, which indicate significant heterogeneity and the random-effect model was implemented, otherwise the fixed-effects model was performed [26]. Using sensitivity analysis of variables, the impact on the overall results by removing one single study was evaluated. Moreover, funnel plots and Egger’s linear regression test were applied for the investigation of publication bias [27]. Possible sources of heterogeneity were examined through univariate and multivariate meta-regression analyses and verified by Monte Carlo Simulation [28, 29].

## Results

### Characteristics of included studies

Our search strategy initially identified 138 articles. By reviewing the titles and abstracts, 67 articles were excluded. After systematically reviewing the remaining full texts, we excluded another 55 articles. In addition, 5 studies were excluded for lack of data integrity. Finally, 11 clinical cohort studies containing a total of 411 patients with CHD met the inclusion criteria used for qualitative data analysis [30, 22, 23, 31–38]. The publication years of eligible studies were between 2002 and 2013. Overall, 9 studies were based on Asians, and the other 2 studies on Caucasians. The NOS score of each included studies was ≥ 5 (moderate-high quality). The characteristics of eligible studies are summarized in Table 1.

**Table 1:** Main characteristics of included studies

First author	Year	Ethnicity	Case number		Gender (M/F)		Age (years)		Study design
			On-pump	Off-pump	On-pump	Off-pump	On-pump	Off-pump	
van Boven WJ [30]	2013	Caucasians	10	10	9/1	8/2	73.3 ± 1.4	73.1 ± 2.2	RCT
Bayram H [22]	2013	Asians	40	24	31/9	18/6	61.9 ± 9.4	60.4 ± 11.3	NON-RCT
Tian LQ [29]	2012	Asians	25	25	-	-	-	-	RCT
Zhai YJ [31]	2008	Asians	10	8	7/3	6/2	60.2 ± 8.5	57.3 ± 6.6	NON-RCT
Hong T [35]	2008	Asians	15	15	11/4	12/3	72.4 ± 4.3	71.8 ± 5.0	NON-RCT
Liu JT [34]	2007	Asians	25	35	-	-	64.3 ± 9.1	65.1 ± 10.3	NON-RCT
Hong F [36]	2007	Asians	15	15	11/4	9/6	58.2 ± 7.2	57.9 ± 6.8	NON-RCT
Bonacchi M [23]	2006	Caucasians	24	18	17/7	12/6	63.5 ± 7.8	63.7 ± 5.4	RCT
Guo XY [37]	2005	Asians	20	20	20/0	20/0	56.8 ± 5.8	58.2 ± 6.5	NON-RCT
Gao CQ [38]	2003	Asians	20	20	17/3	16/4	64.0 ± 8.7	59.0 ± 10.0	RCT
Yan XZ [32]	2002	Asians	9	8	9/0	8/0	63.5 ± 12.1	62.4 ± 10.2	RCT

*M* Male, *F* Female, *RCT* Randomized controlled trial

### Quantitative data synthesis

**Fig. 1:**
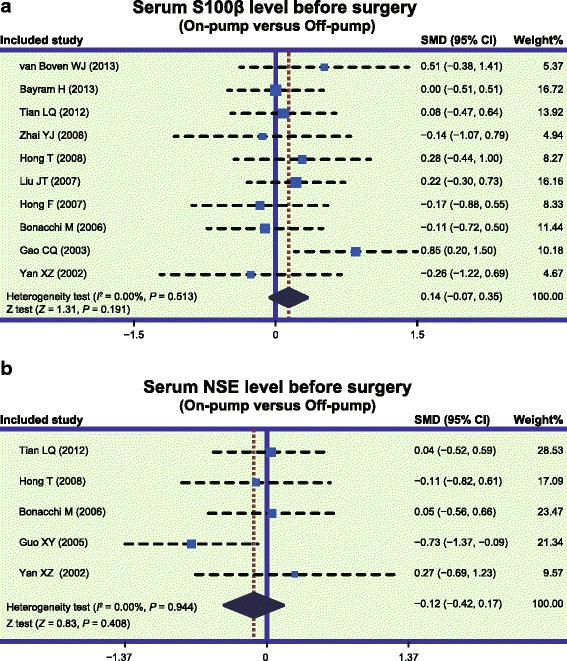
Forest plots for the differences in serum S-100 beta (S-100β) and neuron specific enolase (NSE) levels between on-pump and off-pump groups before surgery (**a**: S-100β; **b**: NSE; SMD: standardized mean difference; CI: confidence interval)

Our meta-analysis showed no significant difference in serum S-100β and NSE levels between the on-pump group and the off-pump group before surgery (S-100β: SMD = 0.14, 95 % CI = −0.07 ~ 0.35, *P* = 0.191; NSE: SMD = −0.12, 95 % CI = −0.42 ~ 0.17, *P* = 0.408; respectively) (Fig. 1).

**Fig. 2:**
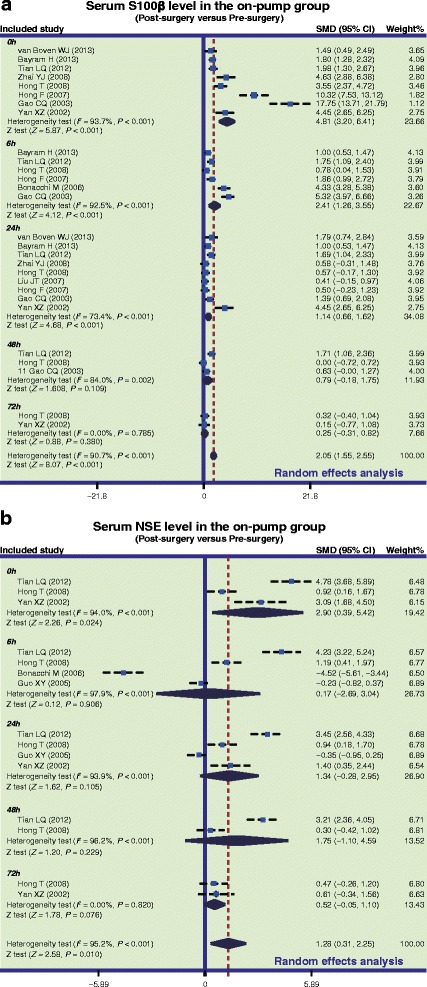
Forest plots for the differences in serum S-100 beta (S-100β) and neuron specific enolase (NSE) levels between before and after surgery in the on-pump and off-pump groups (**a**: S-100β; **b**: NSE; SMD: standardized mean difference; CI: confidence interval)

**Fig. 3:**
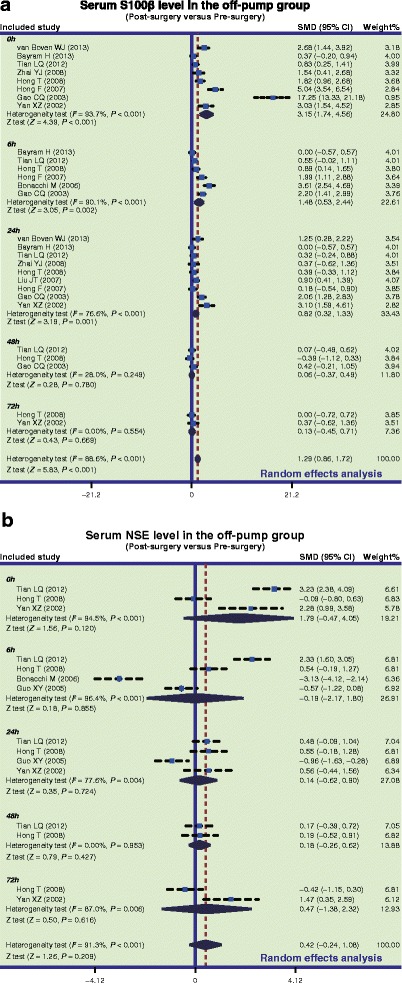
Forest plots for the differences in serum S-100 beta (S-100β) and neuron specific enolase (NSE) levels between before and after surgery in the off-pump groups (**a**: S-100β; **b**: NSE; SMD: standardized mean difference; CI: confidence interval)

**Fig. 4:**
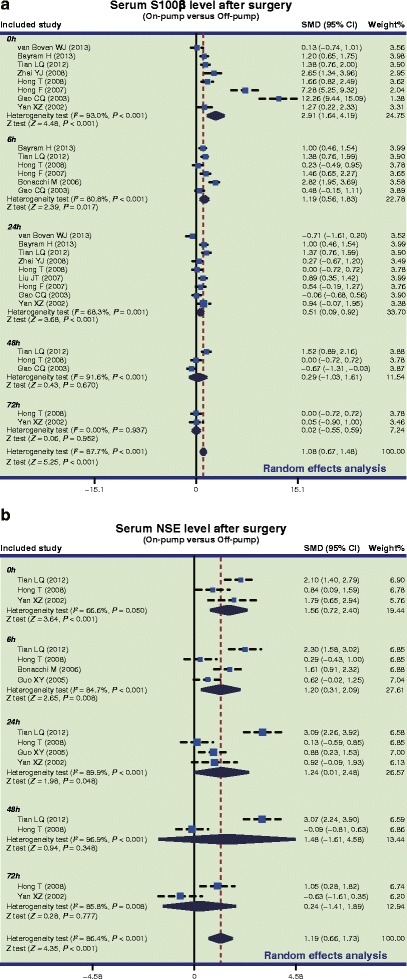
Forest plots for the differences in serum S-100 beta (S-100β) and neuron specific enolase (NSE) levels between on-pump and off-pump groups after surgery (**a**: S-100β; **b**: NSE; SMD: standardized mean difference; CI: confidence interval)

**Fig. 5:**
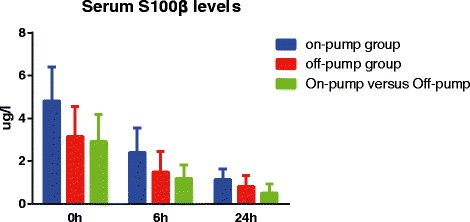
Box-whiskers plots for the differences in serum S-100 beta (S-100β) levels within 24 hours aftersurgery

In the on-pump group, there was a significant difference in serum S-100β levels of CHD patients between before and after surgery (SMD = 2.05, 95 % CI = 1.55 ~ 2.55, *P* < 0.001), especially within 24 h after surgery (0 h: SMD = 4.81, 95 % CI = 3.20 ~ 6.41, *P* < 0.001; 6 h: SMD = 2.41, 95 % CI = 1.26 ~ 3.55, *P* < 0.001; 24 h: SMD = 1.14, 95 % CI = 0.66 ~ 1.62, *P* < 0.001), while no such difference was found after 24 h post-surgery (48 h: SMD = 0.79, 95 % CI = −0.18 ~ 1.75, *P* = 0.109; 72 h: SMD = 0.25, 95 % CI = −0.31 ~ 0.82, *P* = 0.380) (Fig. 2a). In the off-pump group, there was a significant difference in serum S-100β levels between before and after surgery (SMD = 1.29, 95 % CI = 0.86 ~ 1.72, *P* < 0.001), especially within 24 h after surgery (0 h: SMD = 3.15, 95 % CI = 1.74 ~ 4.56, *P* < 0.001; 6 h: SMD = 1.48, 95 % CI = 0.53 ~ 2.44, *P* = 0.002; 24 h: SMD = 0.82, 95 % CI = 0.32 ~ 1.33, *P* = 0.001); however, there was no significant difference observed after 24 h (48 h: SMD = 0.06, 95 % CI = −0.37 ~ 0.49, *P* = 0.780; 72 h: SMD = 0.13, 95 % CI = −0.45 ~ 0.71, *P* = 0.669) (Fig. 3a). Also, our results demonstrated that the serum S-100β of CHD patients in the on-pump group were significantly higher than those of patients in the off-pump group (SMD = 1.08, 95 % CI = 0.67 ~ 1.48, *P* < 0.001), especially within 24 h after surgery (0 h: SMD = 2.91, 95 % CI = 1.64 ~ 4.19, *P* < 0.001; 6 h: SMD = 1.19, 95 % CI = 0.56 ~ 1.83, *P* = 0.017; 24 h: SMD = 0.51, 95 % CI = 0.09 ~ 0.92, *P* = 0.001); after 24 h, the results revealed no such statistical significance (48 h: SMD = 0.29, 95 % CI = −1.03 ~ 1.61, *P* = 0.670; 72 h: SMD = 0.02, 95 % CI = −0.55 ~ 0.59, *P* = 0.952) (Fig. 4a). The difference of the serum S-100β levels between on-pump and off-pump groups was the most significant 0 h after surgery, after which the difference was decreased with time (Fig. 5). According to univariate meta-regression analyses, time may be a source of heterogeneity (*P* = 0.016), while publication year, ethnicity and sample size did not cause heterogeneity (all *P* > 0.05), which was also verified by the multivariate analyses (Table 2).

**Table 2:** Univariate and multivariate meta-regression analyses of potential source of heterogeneity

Heterogeneity factors	Serum S100β levels						Serum NSE levels					
	Coefficient	SE	*t*	*P*	95 % CI		Coefficient	SE	*t*	*P*	95 % CI	
					LL	UL					LL	UL
Publication year												
Univariate	−0.078	0.114	−0.68	0.501	−0.313	0.157	0.189	0.068	2.78	0.016	0.042	0.335
Multivariate	−0.126	0.108	−1.17	0.529	−0.349	0.097	0.125	0.074	1.68	0.336	−0.040	0.290
Ethnicity												
Univariate	−0.583	1.407	−0.41	0.682	−3.474	2.308	0.451	1.165	0.39	0.705	−2.067	2.968
Multivariate	−0.574	1.294	−0.44	0.983	−3.252	2.103	0.050	0.936	0.05	1.000	−2.035	2.136
Time												
Univariate	−0.801	0.311	−2.58	0.016	−1.439	−0.163	−0.219	0.223	−0.98	0.344	−0.702	0.263
Multivariate	−0.896	0.327	−2.74	0.016	−1.571	−0.220	−0.086	0.178	−0.48	0.964	−0.483	0.312
Sample size												
Univariate	0.647	0.866	0.75	0.462	−1.133	2.427	1.408	0.442	3.18	0.007	−0.452	2.364
Multivariate	0.649	0.792	0.82	0.807	−0.990	2.287	0.970	0.534	1.82	0.265	−0.220	2.161

*SE* Standard error. 95 % CI: 95 % confidence interval. *NSE* Neuron specific enolase. *UL* Upper limit. *LL* Lower limit

Furthermore, in the on-pump group, there was a significant difference in serum NSE levels of CHD patients between before and after surgery (SMD = 1.28, 95 % CI = 0.31 ~ 2.25, *P* = 0.010), particularly at 0 h after surgery (SMD = 2.90, 95 % CI = 0.39 ~ 5.42, *P* = 0.024), while it was not significant at other time points (6 h: SMD = 0.17, 95 % CI = −2.69 ~ 3.04, *P* = 0.906; 24 h: SMD = 1.34, 95 % CI = −0.28 ~ 2.95, *P* = 0.105; 48 h: SMD = 1.75, 95 % CI = −1.10 ~ 4.59, *P* = 0.229; 72 h: SMD = 0.52, 95 % CI = −0.05 ~ 1.10, *P* = 0.076) (Fig. 2b). Nevertheless, we found no difference in serum NSE levels before and after off-pump CABG surgery (SMD = 0.42, 95 % CI = −0.24 ~ 1.08, *P* = 0.209) (Fig. 3b). Also, our results demonstrated that the NSE levels of CHD patients in the on-pump group were significantly higher than those of patients in the off-pump group (SMD = 1.19, 95 % CI = 0.66 ~ 1.73, *P* < 0.001), especially within 24 h after surgery (0 h: SMD = 1.56, 95 % CI = 0.72 ~ 2.40, *P* < 0.001; 6 h: SMD = 1.20, 95 % CI = 0.31 ~ 2.09, *P* = 0.008; 24 h: SMD = 1.24, 95 % CI = 0.01 ~ 2.48, *P* = 0.048), but no such difference was found after 24 h (48 h: SMD = 1.48, 95 % CI = −1.61 ~ 4.58, *P* = 0.348; 72 h: SMD = 0.24, 95 % CI = −1.41 ~ 1.89, *P* = 0.777) (Fig. 4b). Based on univariate meta-regression analyses, time, publication year, ethnicity and sample size were all not sources of heterogeneity (all *P* > 0.05), which was further verified by the multivariate analyses (Table 2).

### Sensitivity analysis and publication bias

**Fig. 6:**
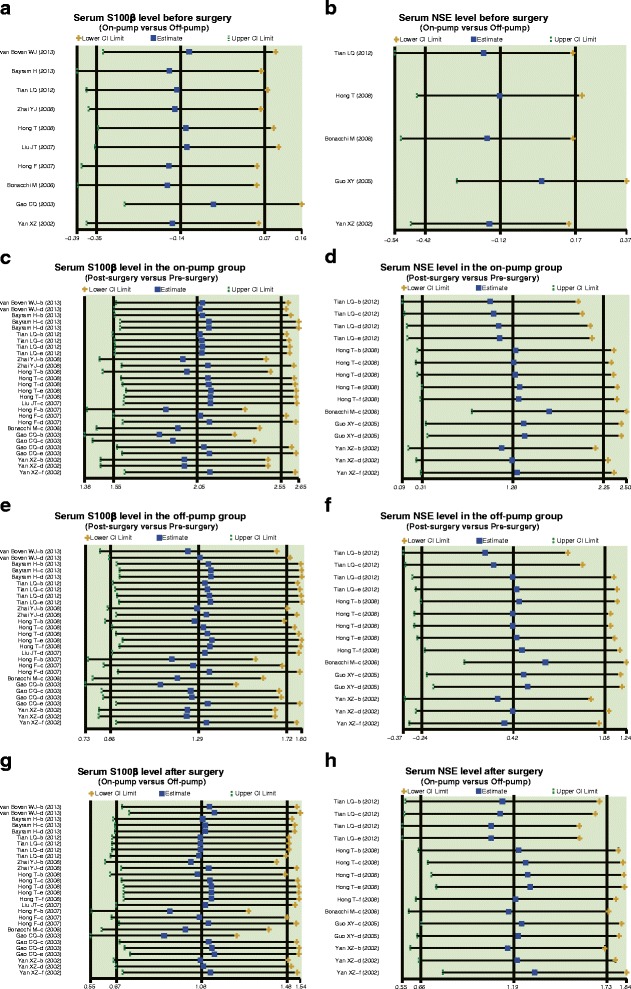
Sensitivity analyses to evaluate the impact of removing one single study on the overall results. **a**: Serum S100ß level before surgery (On-pump versus Off-pump); **b**: Serum NSE level before surgery (On-pump versus Off-pump); **c**: Serum S100ß level in the on-pump group (Post-surgery versus Pre-surgery); **d**: Serum NSE level in the on-pump group (Post-surgery versus Pre-surgery); **e**: Serum S100ß level in the off-pump group (Post-surgery versus Pre-surgery); **f**: Serum NSE level in the off-pump group (Post-surgery versus Pre-surgery); **g**: Serum S100ß level after surgery (On-pump versus Off-pump); **h**: Serum NSE level after surgery (On-pump versus Off-pump)

**Fig. 7:**
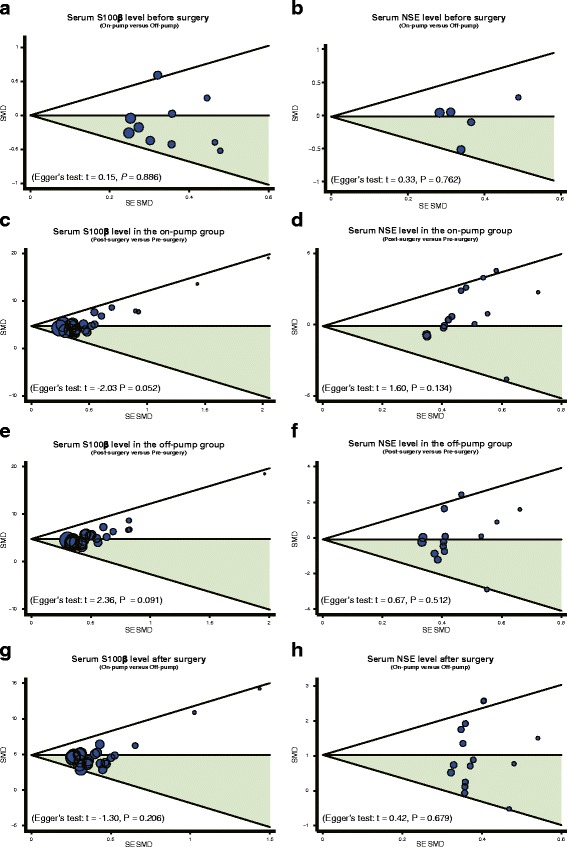
Funnel plots for the differences in serum S-100 beta (S-100ß) and neuron specific enolase (NSE) levels before and after on-pump versus off-pump coronary artery bypass graft surgeries. **a**: Serum S100ß level before surgery (On-pump versus Off-pump); **b**: Serum NSE level before surgery (On-pump versus Off-pump); **c**: Serum S100ß level in the on-pump group (Post-surgery versus Pre-surgery); **d**: Serum NSE level in the on-pump group (Post-surgery versus Pre-surgery); **e**: Serum S100ß level in the off-pump group (Post-surgery versus Pre-surgery); **f**: Serum NSE level in the off-pump group (Post-surgery versus Pre-surgery); **g**: Serum S100ß level after surgery (On-pump versus Off-pump); **h**: Serum NSE level after surgery (On-pump versus Off-pump)

Results of sensitivity analyses indicated that all the included publications had no significant influence on SMD (Fig. 6). Funnel plots revealed no obvious asymmetry (Fig. 7). Also, Egger’s test didn’t illustrate strong statistical evidence of publication bias (all *P* > 0.05).

## Discussion

The present meta-analysis was identified the influence of off-pump and on-pump CABG surgeries on serum levels of S-100β and NSE in patients with CHD. The findings revealed no significant difference in preoperative serum S-100β and NSE levels between off-pump and on-pump CABG groups. The S-100β and NSE proteins cannot be detected in the serum under normal circumstances; however, they can be detected in serum following traumatic cerebral injury, stroke and cardiopulmonary bypass surgery due to impairment of blood–brain barrier (BBB) [39]. We presume that the lack of significant difference in serum S-100β and NSE protein levels before surgery indicates an intact BBB in the patients. Previous evidence showed a positive correlation between serum levels of S-100β and NSE and the neurocognitive dysfunction, because increased S-100β and NSE proteins could leak out from structurally damaged nerve cells into cerebrospinal fluid and secondarily across the BBB [40]. Interestingly, in previous studies the time of cardiopulmonary bypass has been proved strongly correlated with the peak release of S-100β and NSE, and the restrictive fluid management may reduce perioperative cerebral injury [23, 30].

The results also showed that the postoperative serum S-100β and NSE levels were markedly elevated in the on-pump group, especially within 24 h after surgery. Cerebral damage remains one of the major problems associated with open-heart surgery and the contribution of on-pump CABG to cerebral damage is still only partially understood. We hypothesized that during extracorporeal circulation in on-pump CABG, blood and its constituents are likely in contact with foreign surfaces, which may activate inflammation, potentially leading to respiratory insufficiency and damage to lung and brain [22]. Furthermore, brain damage may cause disruption of BBB, which may induce dilatation of small capillaries and arterioles in the brain and then both S-100β and NSE protein may be allowed to release from cerebrospinal fluid to blood fluid in the patients [41]. Thereby, serum levels of S-100β and NSE may increase markedly after on-pump CABG surgery, confirmed by our results, further suggesting that perioperative care should be modified accordingly to control such adverse effects. Additionally, another mechanism of neurocognitive dysfunction in patients undergoing on-pump CABG is cerebral microembolization, which mostly generates from pump circuits and is partially related to the manipulation and instrumentation of the heart using surgical instrumentation, especially the aorta [42]. These embolic events can also result in increased serum S-100β and NSE levels postoperatively in patients [43].

We found a significant difference in the serum S-100β levels before and after off-pump CABG surgery, while no significant difference in serum NSE levels were observed in the off-pump group before and after surgery. In a previous study, the peak release of S-100β occurs at 6 h postoperatively and signifies perioperative brain damage, while the NSE peak serum levels occurred beyond 24 h after the surgery in patients undergoing off-pump CABG [44, 45]. Therefore, the different peak times of the peak serum S-100β and NSE levels may be a the reason that significant changes are observed in S-100β levels and not in NSE levels before and after off-pump CABG. Bonacchi et al. also found that in the off-pump group, serum levels of S-100β and NSE were almost within the normal range preoperatively; but only the S-100β serum levels increased significantly postoperatively [23].

Another principal finding in our meta-analysis is that postoperative serum S-100β and NSE protein levels were significantly higher in the on-pump group than those in the off-pump group, especially within 24 h after surgery, implying that off-pump CABG may be associated with lower risk of neurocognitive dysfunction than on-pump CABG. Although the precise mechanism through which off-pump CABG reduces systemic inflammation in brain damage and postoperative mortality is still not fully understood, it may be reasonable to postulate that off-pump CABG and decrease the frequency of cerebral embolism [46]. Huseyin Bayram et al. has showed that the postoperative serum S-100β levels in the off-pump group were significantly lower than that in the on-pump CABG group [22]. Similarly, Lee et al. have observed that off-pump CABG surgery may decrease neurological and clinical morbidity in comparison to on-pump CABG in a randomized group of 60 patients undergoing on-pump and off-pump procedures and complemented by neurocognitive testing before surgery and 2 week/1 year after surgery [47]. By contrast, Edwards compared on-pump and off-pump CABG with a year of follow-up study, reporting that on-pump CABG is superior to off-pump CABG, although off-pump CABG had advantages of time on mechanical ventilation, bleeding and need for reoperation etc. [48]. Despite these contradictory results on whether off-pump CABG is superior to the on-pump CABG [49], our results are in accordance with several studies that demonstrated that the preoperative brain injury evaluated by the release of NSE and S-100β protein is significantly higher in patients undergoing off-pump CABG than patients receiving on-pump CABG. Our study has limitations which should be interpreted. First, through searching the databases, only 5 randomized controlled trials relevant to the topic were identified (the other 6 studies were non-randomized controlled trials), which may cause bias due to the small sample size. Second, because all the included randomized controlled trials could not demonstrated any significant impairment of cognitive function after both on-pump and off-pump surgeries, these studies are more likely to provide “academic” rather than clinical evidence, therefore future clinical evidence are needed. Third, the meta-analysis could not acquire the original data and information on the techniques used in the surgeries was limitedly provided in the studies included, which may restrict further evaluation of the plausible effect of off-pump and on-pump CABG on serum S-100β and NSE levels. Moreover, Due to the lack of data on neurological complications in the enrolled studies, we failed to identify a relationship between higher marker levels and neurological events. Even though there are several limitations, our study is the first meta-analysis on the comparison of serum levels of S-100β and NSE between patients treated with on-pump and off-pump CABG. More importantly, a literature search strategy with high sensitivity was implemented for electronic databases. In order to identify other potential articles, we also manually searched the reference lists of relevant articles, and the eligible articles were selected on the basis of strict inclusion and exclusion criteria. Besides, pooling of information from each study is founded on rigorous statistical analysis.

## Conclusions

Our findings revealed that off-pump and on-pump CABG surgeries may increase serum S-100β and NSE levels in CHD patients, especially within 24 h of on-pump CABG surgery. However, more researches with more detailed data and large sample size are necessary to confirm our findings and validate the clinical use of S-100β and NSE as reliable biomarkers to predict outcomes.

## Abbreviations

CABG: Coronary artery bypass graft
CHD: Coronary heart disease
NSE: Neuron specific enolase
CBM: Chinese Biomedical Database
NOS: The Newcastle-Ottawa Scale
SMD: Standardized mean difference
CI: Confidence intervals
BBB: Blood–brain barrier
